# Cardiac Arrest Due to Lymphocytic Colitis

**DOI:** 10.7759/cureus.87757

**Published:** 2025-07-11

**Authors:** Natraj Hurdyal

**Affiliations:** 1 Emergency Department, Leighton Hospital, Crewe, GBR

**Keywords:** automatic cardioverter defibrillator, bowel ischemia and colitis, loose stools, out of hospital cardiac arrest, post cardiac arrest care, serum potassium level

## Abstract

The case report presents a patient with a history of chronic watery diarrhoea, which was present five months before the cardiac arrest. Her health history suggested she experienced lymphocytic colitis, and the physicians administered budesonide. The physician defibrillated the patient twice. Defibrillation involves the use of a machine that sends electric signals to the heart to help it regain its function. It is used immediately after a patient arrives at the emergency department to reduce organ damage and save an individual’s life. After stabilisation, the patient was referred to the gastroenterology department. In the case presented, the patient experienced a heart attack as a result of hypokalemia.

## Introduction

Cardiac arrest is a common public health crisis in the UK requiring emergency medical care. In the UK, about 60,000 cardiac arrests occur annually in individuals out of the hospital [1]. Statistics indicate that 72% of these cases occur at home [1]. Additionally, only 11% of patients discharged from hospital after treatment survive [2]. This case study provides important insights into patient experiences and how existing treatment approaches may help address the problem. Additionally, case studies provide a comprehensive exploration of the medical condition to inform clinical practice, enhance medical education and provide background for the development of better interventions. The aim of this report is to present a case of cardiac arrest due to hypokalemia associated with lymphocytic colitis.

## Case presentation

Timeline of events

A 67-year-old African American woman experienced a sudden cardiac arrest at her general practitioner. The physician started cardiopulmonary resuscitation (CPR) including chest compressions and ventilations immediately to restore her breathing condition. The physician alerted the emergency medical services, and an ambulance arrived six minutes later. Ventricular fibrillation was the initial cardiac rhythm. The physician defibrillated the patient eight times and an organised rhythm was noted several times afterwards. The medical team administered lidocaine, atropine, amiodarone, and adrenaline.

**Figure 1:**
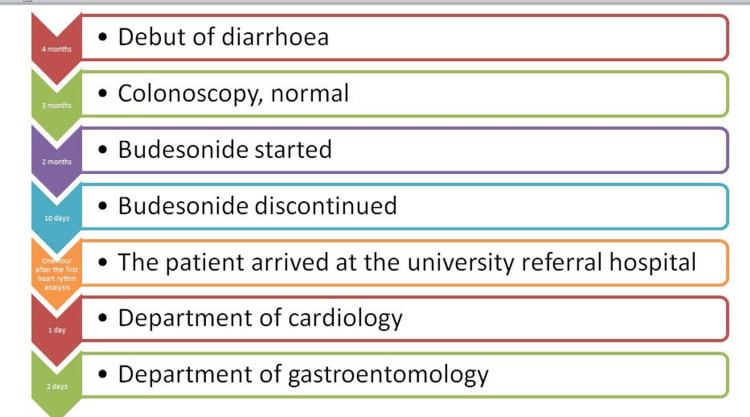
Timeline of Events

The patient arrived at the university referral hospital one hour after the first heart rhythm analysis as shown in Figure 1. The patient had a history of chronic watery diarrhoea, which presented five months before the cardiac arrest. Initially, she reported continuous diarrhoea without warning signs, affecting her ability to perform her daily activities. A colonoscopy was performed, and the findings indicated that she had a normal macroscopic appearance. Her health history suggested she experienced lymphocytic colitis, and the physicians administered budesonide. At the time of diagnosis, the patient indicated that she had used acetylsalicylic acid. The physician recommended that she stop using the medication as she had a history of stroke. Figure 1 shows the timeline of events.

Findings and management

Upon arrival at the receiving hospital, clinicians immediately began CPR using LUCAS® 2 Chest Compression System (Jolife, part of Stryker, Lund, Sweden). The physician defibrillated the patient twice. After performing a coronary angiography, doctors found no signs of coronary artery disease. An echocardiography also showed no signs of structural heart disease. However, further tests showed a potassium level of 2.0 mmol/L, which is below the normal range of 3.5 to 4.6 mmol/L for adults. Additionally, the pH level was measured at 6.86, which is lower than normal. Table 1 shows the contrast between the findings and the reference ranges.

**Table 1:** Potassium and pH Tests

	Findings	Reference Range
Potassium Level	2.0 mmol/L	3.5 to 4.6 mmol/L
pH	6.86	7.37 to 7.45

The emergency team noted a recurrence of ventricular tachyarrhythmias and admitted the patient to the intensive care unit (ICU). The physicians defibrillated her a further five times. The propensity of the ventricular tachyarrhythmias ceased as the medical team corrected her serum potassium level. The patient showed significant improvement after 24 hours and was transferred to the cardiac care ward. The nurses did not observe arrhythmias in the next 24 hours. The patient was transferred to the gastroenterology for further assessment, treatment and ultimately discharge. A neurologist assessed her three months after discharge from the department and found her neurologically healthy. 

The patient indicated that she had visited the gastroenterology outpatient clinic 10 days before the cardiac arrest because budesonide showed a lack of effect. The physician at the clinic discontinued the glucocorticoid and recommended psyllium husk. Previously, the patient had shown improvement through this intervention. Additionally, the physician found that her potassium level was normal in the first two and a half months after experiencing chronic diarrhoea. However, the physician did not monitor the potassium level for the next six weeks before she experienced cardiac arrest.

## Discussion

The inflammatory nature of colitis suggests that this might be a primary cause in this particular case, but we should not neglect the contributing factor of the previous history of stroke. According to the American Heart Association [2], the new onset of heart-related complications in a stroke patient is a major clinical challenge and in this particular case, this patient had ventricular arrhythmia and cardiac arrest. “The severity of histological change is most prominent in the proximal colon and declines distally; that is, biopsies from the right or transverse colon are optimal” [1]. The condition differs from lymphocytic colitis and collagenous colitis in histopathology. Individuals aged between 55 and 70 years are at the highest risk of developing the condition. Additionally, it is more common in women than men at a ratio of 3:1 [2]. In most cases, the cause of the disease is unknown. However, evidence shows that certain medications may induce the condition.

Multiple studies indicate that nonsteroidal anti-inflammatory drugs and acetylsalicylic acid are the most common causes of lymphocytic colitis [2,3]. Other medications that have been linked to the condition include simvastatin omeprazole flutamide, ticlopidine, sertraline, ranitidine, and acarbose. Evidence indicates that 10%-20% of all cases of chronic watery diarrhoea lead to a microscopic colitis diagnosis [3]. The increased secretion and decreased absorption of chloride and sodium ions are believed to be the diarrhoea-causing mechanism in collagenous colitis [2]. Unabsorbed anions and secondary hyperaldosteronism are responsible for the increase in faecal potassium losses among individuals with diarrhoea.

It is critical that the patient's general physician keeps this condition in mind. Their responsibility should include the evaluation of their current medications as prescribed by other providers. The physician evaluates the patient’s current medications and diet factors that may lead to gut problems and diarrhoea. Medications that increase the risk of lymphocytic colitis must be stopped [3]. Additionally, the physician recommends that the patient reduce the intake of alcohol, caffeine, and dairy products. “If symptoms are debilitating, budesonide, a glucocorticoid with low systemic effect due to its substantial elimination by first-pass hepatic metabolism, has been proven effective” [1]. Multiple studies suggest that diarrhoea may stop within a few weeks after treatment [2,3]. Additionally, positive results have been observed among patients who do not take any treatment for diarrhoea.

In the case presented, the patient experienced a cardiac arrest as a result of hypokalemia. More specifically, hypokalemia develops due to diarrhoea associated with microscopic colitis. Evidence suggests that budesonide treatment contributes to hypokalemia. The patient in the case study stopped this medication 10 days before the heart attack. After treatment for cardiac arrest, the patient resumed the medication. Additionally, diarrhoea stopped without any impact on potassium levels. Multiple tests were carried out to rule out other possible causes of the emergency situation. The physician could not prove any other cause of cardiac arrest after resuscitation.

There are low chances of surviving a cardiac arrest out of the hospital [3]. “The interventions linking a patient with cardiac arrest to survival with a good cerebral outcome are known as the chain of survival” [4]. These interventions may include early diagnosis, early advanced life support, early defibrillation and early bystander CPR. Patients show positive outcomes after effective chest compression with the right compression frequency and depth. Most patients experience poor outcomes due to delays in receiving care. Additionally, physicians may fail to determine the primary cause of the cardiac arrest, affecting their ability to develop an effective treatment plan.

Defibrillation involves the use of a machine that sends electric signals to the heart to help it regain its function. It is used immediately after a patient arrives at the emergency department to reduce organ damage and save an individual’s life [2]. However, multiple studies indicate that surgery may be needed in cases where defibrillation is ineffective. Generally, this surgery involves placing a certain device in the heart to help it improve its ability to pump blood [3]. The physicians run multiple tests in the emergency room to determine the root cause of the heart attack. The findings help them develop an effective treatment plan.

One of the most common procedures for heart conduction abnormalities is an implantable cardioverter-defibrillator (ICD). It involves placing a battery-powered device under the skin around the collarbone. It checks the heart rhythm continuously and sends shock waves to reset the heart conditions in emergency situations. Multiple studies indicate that ICD may stop life-threatening changes in the heart rhythm [1]. Coronary artery bypass surgery is another procedure performed on patients experiencing heart attacks. It creates a new pathway to allow blood to flow past the blocked artery [5]. The procedure allows the heart to receive adequate blood to perform its functions effectively, reducing the risk of future heart attacks.

Some physicians may opt for coronary angioplasty to open clogged or blocked arteries in the heart. Physicians need to conduct multiple tests to identify blocked arteries in the heart. During the procedure, the physician inserts a flexible tube into the artery and moves it slowly to the blocked area [2]. The tube contains a tiny balloon, which is widened upon reaching the narrowed area of the blood vessel. The balloon expands the blood vessels, allowing blood to flow to the heart. The physician may pass a stent through the tube to help keep the blood vessel open.

Multiple studies indicate that heart attacks may be prevented through lifestyle modifications and training. Individuals at the risk of heart attack need to stop alcohol consumption and avoid smoking [4]. Additionally, they need to manage their stress and consume a healthy diet. Also, multiple studies recommend that cardiac arrest patients need to engage in physical activity to improve their blood flow [6]. Families with cardiac arrest patients need to be trained on how to perform CPR in emergency situations. This training may help prevent death before patients reach the hospital for specialised treatment [7]. The American Red Cross and other non-governmental organisations provide courses in defibrillator and CPR use.

## Conclusions

The case report presents a patient with a history of chronic watery diarrhoea, which was present five months before the cardiac arrest. Her health history suggested she experienced lymphocytic colitis, and the physicians administered budesonide. The physician defibrillated the patient twice. After stabilisation, the patient was referred to the gastroenterology department. In the case presented, the patient experienced a cardiac arrest as a result of hypokalemia. On the other hand, hypokalemia develops due to diarrhoea associated with microscopic colitis. Evidence suggests that budesonide treatment contributes to hypokalemia. The patient in the case study stopped this medication 10 days before the heart attack. After treatment for cardiac arrest, the patient resumed the medication. The case emphasises the importance of treating the underlying cause of cardiac arrest to improve a patient’s health outcome.
